# Effect of Aqua Points Level on Speed, Efficiency, and Turn Performance in Youth Swimmers Aged 11–12 Years

**DOI:** 10.3390/jfmk11030278

**Published:** 2026-07-18

**Authors:** Alejandro López-Hernández, José Francisco Alonso Ramos, Juan Ángel Simón Piqueras, Miriam De la Torre Maroto, José María González Ravé

**Affiliations:** 1 Sport Training Laboratory, Faculty of Sports Sciences, University of Castilla-La Mancha, 45071 Toledo, Spain; fran.alonso@alu.uclm.es; 2 Faculty of Education of Albacete, University of Castilla-La Mancha, 02071 Albacete, Spain; juanangel.simon@uclm.es; 3 Castilla-la-Mancha Swimming Federation, Castilla-la-Mancha, 02001 Albacete, Spain; miriamla.torre@hotmail.com

**Keywords:** youth swimming, Aqua Points, biomechanics, turn performance, swimming efficiency, competitive level, field-based assessment

## Abstract

**Background:** This study aimed to identify the field-based technical and biomechanical variables that best discriminate and predict competitive performance in 11–12-year-old swimmers. **Methods:** Seventy-two federated swimmers were assessed during a controlled technical evaluation session conducted four weeks prior to the Under-13 (U–13) Regional Championship. Variables measured included 25 m freestyle time and velocity, 25 m kick time and velocity, turn time and velocity using the 5 m in–5 m out criterion, stroke count, stroke length, and swimming efficiency index. Aqua Points were obtained from official championship results held one month after testing. Swimmers were categorized into three performance groups according to Aqua Points classification: low, medium, and high competitive level. A multiple linear regression analysis was performed to identify predictors of competitive performance. **Results:** Significant differences were observed between competitive levels for 25 m freestyle time and velocity, kick performance, turn time and velocity, and swimming efficiency (*p* < 0.05), with progressive improvements as Aqua Points increased. Significant differences were also observed for stroke count and stroke length, suggesting that stroke mechanics contributed to performance differentiation within this age group. In the regression analysis, 25 m freestyle velocity and turn velocity emerged as the strongest predictors of competitive performance, while sex was a significant covariate. The final model explained a large proportion of the variance in Aqua Points (*R*^2^ = 0.846, adjusted *R*^2^ = 0.832, *p* < 0.001). **Conclusions:** Competitive level in 11–12-year-old swimmers is strongly associated with technical proficiency, particularly short-distance swimming speed and turn execution. These findings highlight the importance of technical development during formative stages and provide practical insights for coaching strategies.

## 1. Introduction

Swimming performance is determined by the complex interaction of physiological, biomechanical, and technical factors [1,2,3]. From a mechanical perspective, swimming velocity depends on the balance between propulsive forces and hydrodynamic drag [4]. Therefore, the ability to generate effective propulsion while minimizing both active and passive drag has consistently been identified as a key determinant of performance [2,5].

In this context, biomechanical and technical variables provide relevant information for understanding why some swimmers achieve higher competitive performance than others.

Among these determinants, sprint swimming speed, propulsion capacity, stroke mechanics, and turn execution are particularly relevant in competitive swimming. Recent evidence has highlighted the importance of neuromuscular force production for in-water performance, with dry-land force-velocity characteristics being associated with race performance across different strokes and distances [6].

These findings suggest that greater force production capacity may enhance propulsive effectiveness, particularly in sprint events. In addition, turn performance has been identified as a critical component of race success, particular in short-course events [7,8,9,10]. Efficient turns reduce deceleration during wall approach and maximize propulsion during push-off and underwater phases, making turn execution an important technical determinant of performance.

The relevance of these biomechanical and technical factors may be even greater in youth swimmers. Between 11 and 12 years of age, athletes experience rapid neuromuscular development and enhanced motor learning capacity [11,12]. At this stage, improvements in performance are typically driven more by coordination and technique than by physiological adaptations [13,14]. Consequently, the assessment of technical variables such as stroke mechanics, propulsion, and efficiency becomes particularly relevant.

However, it is also important to recognize that swimmers of the same chronological age may differ in their biological maturation status, which can influence body size, muscle strength, propulsion capacity, and overall swimming performance. Consequently, developmental variability should be considered when interpreting performance differences in preadolescent swimmers, as biological maturation may modulate the relationship between biomechanical characteristics and competitive performance [15,16].

Despite the importance of technical assessment in youth swimming, limited research has examined how field-based biomechanical variables relate to objective classifications of competitive level. Performance classification systems, such as Aqua Points, allow objective comparison of competitive level across swimmers by standardizing race performance relative to reference benchmarks [17]. Although Rudolph Points are commonly used in youth swimming due to their age-adjusted scoring system, Aqua Points were selected in the present study because they provide an internationally standardized performance metric based on absolute performance. This allows for consistent comparison of competitive level across swimmers and facilitates the analysis of associations with biomechanical variables independently of age-group adjustments.

Understanding these relationships may provide useful information for coaches by identifying the technical variables most strongly associated with competitive performance during a critical stage of swimmer development.

Therefore, the aim of this study was to identify the field-based technical and biomechanical variables that best discriminate and predict competitive performance in youth swimmers aged 11–12 years. Specifically, the study sought to analyze whether swimmers with higher competitive performance demonstrated superior sprint speed, lower-limb propulsion capacity, turn execution, and swimming efficiency during a controlled technical assessment conducted prior to competition.

It was hypothesized that swimmers with higher Aqua Points would exhibit significantly better performance outcomes, characterized by faster 25 m freestyle times, higher swimming and kicking velocities, and more efficient turn execution compared with lower-level swimmers.

Additionally, technical efficiency indicators were expected to differ between competitive levels, whereas structural stroke variables such as stroke count and stroke length were expected to show smaller or non-significant differences. This expectation was based on previous evidence suggesting that performance development in young swimmers is strongly influenced by technical efficiency, coordination, and biomechanical organization [13,14], while stroke mechanics may also be affected by inter-individual differences in growth and maturation-related development [15,16]. Although biological maturation was not directly assessed in the present study, sex and birth quarter were included as covariates in the statistical analysis to partially account for developmental variability and potential relative age-related differences [15,16]. 

## 2. Materials and Methods

### 2.1. Participants

Seventy-two swimmers (35 boys and 37 girls) aged 11–12 years competing at the U–13 regional level participated voluntarily. Participants were recruited from different swimming clubs within the Castilla-La Mancha region (Spain) according to their competitive ranking and attended a single testing session at the same swimming facility.

All swimmers trained 4–6 times per week, had at least two years of competitive experience, and were free from injury at the time of testing. Parental/legal guardian consent and participant assent were obtained. The study was conducted in accordance with the Declaration of Helsinki and approved by the local University Ethics Committee (CEIS-739611-H7M1; approval date: 25 February 2024).

### 2.2. Study Design

This study used a cross-sectional, field-based observational design to identify the field-based technical and biomechanical variables that best discriminate and predict competitive performance in youth swimmers.

All swimmers completed a single standardized testing session conducted four weeks before the U–13 Regional Championship. Biomechanical and performance variables were collected during this session, whereas competitive performance was determined from Aqua Points obtained from each swimmer’s best 50 m freestyle performance at the U–13 Regional Championship held one month later.

For group comparisons, swimmers were classified into low-, medium-, and high-performance groups according to tertiles of the Aqua Points distribution within the sample (low ≤ 224, medium 225–277, high ≥ 278). This approach ensured balanced group sizes and continuous classification without gaps.

### 2.3. Procedures

**Figure 1:**
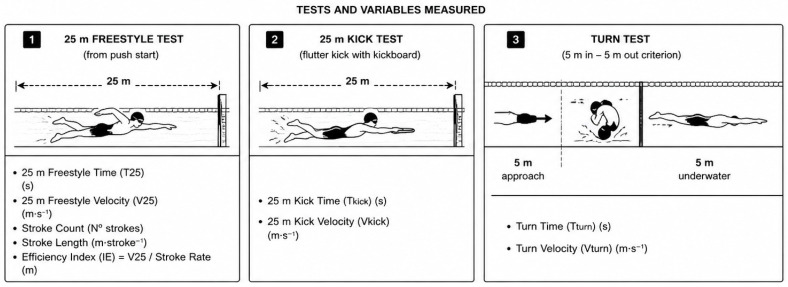
Experimental protocol and variables measured. Swimmers performed a 25 m freestyle test, a 25 m kick test, and a turn test (5 m in–5 m out). Performance variables and biomechanical parameters were recorded and used to predict competitive performance (Aqua Points).

Testing was conducted during a single standardized session performed four weeks before the U–13 Regional Championship in a 25 m indoor swimming pool under controlled conditions (water temperature approximately 27–28 °C). Following a standardized warm-up, swimmers completed three field-based tests in the following order: (i) a maximal 25 m freestyle test, (ii) a maximal 25 m flutter kick test, and (iii) a maximal turn test using the standardized 5 m in–5 m out criterion. Stroke mechanics were assessed by manually counting arm cycles over 25 m using two independent raters, from which stroke length was calculated. To ensure reliability, both raters were previously trained and inter-rater agreement was verified prior to data collection. Swimming efficiency was quantified using a velocity-based efficiency index. The complete testing protocol is illustrated in Figure 1.

#### 2.3.1. Warm-Up

Before testing, all participants completed a standardized 20 min warm-up designed to ensure comparable physiological readiness and technical preparation prior to performance assessment. The warm-up protocol was previously planned by the research team and written on a poolside whiteboard so that all swimmers could clearly follow the sequence of exercises. Evaluators provided a standardized verbal explanation of the warm-up procedure to guarantee that all participants understood the tasks and performed them under identical conditions.

The warm-up began with approximately 5 min of dry-land activation exercises performed on the pool deck, including general mobility movements, joint activation, and dynamic exercises targeting the upper and lower limbs commonly involved in swimming performance. This phase aimed to increase muscle temperature and prepare swimmers neuromuscularly before entering the water.

After dry-land activation, swimmers entered the pool and completed 15 min of in-water warm-up consisting of progressive technical drills and low-to-moderate intensity swimming. The in-water phase included technical exercises focused on body alignment, stroke coordination, and kicking mechanics, gradually increasing intensity toward the end of the warm-up to approximate competition readiness while avoiding fatigue.

Warm-up has been shown to enhance performance through increases in muscle temperature, improved oxygen delivery, and enhanced neuromuscular function [18]. In swimming, combined in-water and dry-land warm-up strategies are commonly recommended to optimize performance and maintain muscle activation prior to maximal efforts [19].

All swimmers performed the same warm-up simultaneously under researcher supervision to ensure protocol standardization and consistency across participants prior to the testing trials.

#### 2.3.2. Testing Order

Maximal 25 m Freestyle (Push Start).

Each swimmer performed a maximal 25 m freestyle trial starting from a push-off against the wall while maintaining their habitual competitive technique. Short maximal swimming trials are widely used to assess sprint performance, swimming-specific speed, and anaerobic power in competitive swimmers, as they reproduce the biomechanical and energetic demands of sprint events under controlled conditions [20,21]. These short-distance maximal efforts allow the evaluation of performance-related variables while minimizing pacing strategies and emphasizing maximal propulsion, stroke effectiveness, and swimming efficiency.

#### 2.3.3. 25 m Kick Drill

Participants completed a 25 m maximal-effort flutter kick trial using a kickboard to isolate lower-limb propulsion while minimizing upper-limb contribution. Kick time was recorded, and kick velocity was calculated as distance divided by time. This test was included to provide a field-based assessment of lower-limb propulsion capacity under standardized conditions, given the recognized contribution of the lower limbs to force production during front crawl swimming [22,23].

#### 2.3.4. Turn Performance (5 m in–5 m Out Criterion)

Turn performance variables were obtained from a separate maximal turn test using the standardized 5 m in–5 m out criterion. This method evaluates performance across a 10 m segment centered on wall contact, including the approach phase, rotation, push-off, underwater phase, and breakout. The selection of this criterion was based on its widespread use in swimming biomechanics research, as it provides a reliable and ecologically valid measure of turn performance while minimizing the influence of free-swimming velocity before and after the wall [8,9,10,24].

Previous biomechanical analyses have demonstrated that swimming turns represent a critical determinant of overall race performance, particularly in sprint and short-course events, where small improvements in wall-contact efficiency and underwater propulsion can substantially influence total race time [8,9,10]. Furthermore, contemporary race analyses at world-class level have consistently adopted the 5 m in–5 m out approach to standardize turn assessment and allow meaningful comparisons between swimmers [10]. A recent systematic review on swimming turn biomechanics also supports the use of this criterion as a methodological reference for research and applied performance analysis [24].

Turn time and turn velocity were therefore calculated across this standardized 10 m section to quantify swimmers’ technical efficiency during direction change and underwater propulsion under competitive-like conditions.

### 2.4. Statistical Analysis

Statistical analyses were performed using IBM SPSS Statistics (Version 26, IBM Corp., Armonk, NY, USA). Data normality was assessed using the Shapiro–Wilk test, and all variables met the assumption of normality (*p* > 0.05), allowing the use of parametric tests.

Homogeneity of variances was verified using Levene’s test prior to inferential analyses. A one-way analysis of variance was conducted to examine differences between performance groups, with Aqua Points classification used as the independent variable. When significant main effects were observed, Bonferroni-adjusted post hoc comparisons were applied. Results are reported including degrees of freedom, *F*-values, and *p*-values. Effect sizes were calculated using partial eta squared (η^2^p).

For the ANOVA, Aqua Points were categorized into three performance groups (low, medium, and high) based on tertiles of the sample distribution and used as the independent variable.

A multiple linear regression analysis was performed using Aqua Points as a continuous dependent variable to identify the technical predictors of competitive performance. Sex (male = 0, female = 1) and birth quarter (Q1–Q4) were included as covariates to account for potential biological differences and Relative Age Effects (RAE).

Prior to regression analysis, the assumptions of linearity, independence of errors, normality of residuals, homoscedasticity, and absence of multicollinearity were assessed. Linearity was examined through scatterplots, independence of errors using the Durbin–Watson statistic, normality of residuals through visual inspection of histograms and Q–Q plots, and homoscedasticity by plotting standardized residuals against predicted values. Multicollinearity was assessed using tolerance and variance inflation factor (VIF) values.

Model fit was evaluated using the coefficient of determination (*R*^2^) and adjusted *R*^2^. Statistical significance was set at *p* < 0.05.

## 3. Results

Descriptive statistics for all performance and biomechanical variables across groups are presented in Table 1. A clear performance gradient was observed, with higher-level swimmers demonstrating faster 25 m times, greater swimming and turn velocities, and higher efficiency values compared to lower-level groups.

**Figure 2:**
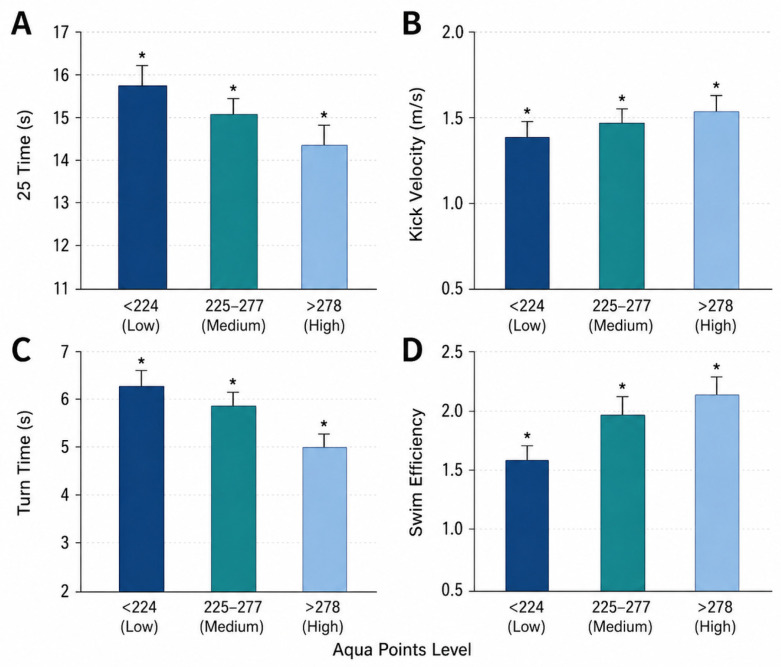
Comparison of performance-related variables between swimmers grouped according to Aqua Points classification: (**A**) 25 m freestyle time, (**B**) kick velocity, (**C**) turn time (5 m in–5 m out), and (**D**) swimming efficiency index. Data are presented as mean ± standard deviation. *: *p* < 0.05 indicates significant differences between groups.

Between-group comparisons revealed significant differences for several performance variables (Figure 2). Specifically, 25 m freestyle time differed significantly between groups, F(2, 43) = 8.91, *p* < 0.001, η^2^p = 0.29, indicating progressively faster sprint performances across increasing Aqua Points tertiles. Kick velocity also differed significantly between groups, F(2, 43) = 11.20, *p* < 0.001, η^2^p = 0.34. Turn time showed the strongest between-group differences, F(2, 43) = 15.97, *p* < 0.001, η^2^p = 0.43, demonstrating a large effect of competitive level on turn execution. In addition, the swimming efficiency index differed significantly across performance levels, F(2, 43) = 6.23, *p* = 0.004, η^2^p = 0.22.

Contrary to the initial hypothesis, significant differences were also observed in stroke count, F(2, 43) = 5.35, *p* = 0.008, η^2^p = 0.20, and stroke length, F(2, 43) = 3.84, *p* = 0.029, η^2^p = 0.15, suggesting that structural stroke characteristics may also contribute to competitive differentiation within this 11–12-year-old cohort.

Analysis of birth distribution across the calendar year showed a slight overrepresentation in the first half (Q1–Q2); however, no significant association was found between birth quarter and Aqua Points (*p* > 0.05).

The regression analysis identified several significant predictors of competitive performance (Table 2).

The stepwise regression analysis retained 25 m freestyle velocity, turn velocity, efficiency index, and sex as significant predictors of competitive performance, while 25 m kick velocity was excluded from the final model.

The final regression model explained a large proportion of the variance in competitive performance (*R*^2^ = 0.846, adjusted *R*^2^ = 0.832), and the overall model was statistically significant (F(4, 67) = 81.30, *p* < 0.001), indicating a high level of explanatory power.

Among the predictors, 25 m freestyle velocity (B = 358.20, *p* < 0.001) and turn velocity (B = 145.65, *p* = 0.001) showed the strongest positive associations with Aqua Points. The efficiency index showed a small but statistically significant negative association (B = −25.04, *p* = 0.028).

Sex was also a significant predictor, with females scoring on average 86.67 Aqua Points higher than males when controlling for the remaining variables (*p* < 0.001).

From an applied perspective, the magnitude of the regression coefficients indicates that a 0.10 m·s^−1^ increase in turn velocity is associated with an estimated improvement of approximately 14.6 Aqua Points, whereas a similar increase in 25 m freestyle velocity corresponds to an improvement of approximately 35.8 Aqua Points, independently of other variables in the model.

**Figure 3:**
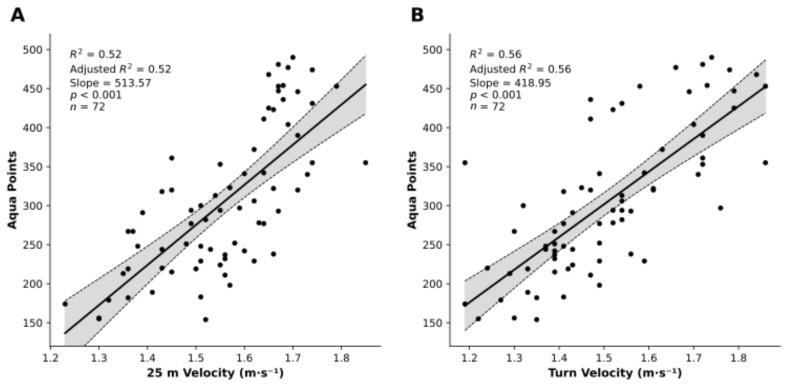
Relationships between key performance variables and competitive performance (Aqua Points). (**A**) Association between 25 m freestyle velocity and Aqua Points. (**B**) Association between turn velocity (5 m in–5 m out) and Aqua Points. Solid lines represent linear regression fits, and shaded areas indicate 95% confidence intervals. Both variables showed strong positive associations with competitive performance.

Figure 3 shows the relationship between 25 m velocity (A) and turn velocity (B) with Aqua Points. Both variables showed strong positive associations with Aqua Points: 25 m velocity explained 52% of the variance (*R*^2^ = 0.52, *p* < 0.001), whereas turn velocity explained 56% of the variance (*R*^2^ = 0.56, *p* < 0.001).

## 4. Discussion

The present study identified the field-based technical and biomechanical variables that best discriminate and predict competitive performance in 11–12-year-old swimmers. The main findings showed that higher-performing swimmers demonstrated superior sprint velocity, turn performance, swimming efficiency, and stroke mechanics. Furthermore, the regression analysis identified 25 m freestyle velocity and turn velocity as the strongest independent predictors of competitive performance.

Lower-limb propulsion also differed significantly between competitive levels, with higher-performing swimmers exhibiting greater kick velocity. This finding agrees with previous evidence highlighting the contribution of the lower limbs to force production and sprint swimming performance [22,23]. However, kick velocity was not retained in the final regression model, suggesting that its influence may be largely mediated through overall swimming velocity and whole-body coordination rather than representing an independent predictor of competitive performance [23].

Turn performance emerged as a critical discriminating factor between competitive levels, with higher-performing swimmers demonstrating faster turn times and greater turn velocities. These results are in agreement with previous studies emphasizing the importance of non-cyclic race segments in sprint performance [8,9,10,24]. Efficient turns reduce velocity loss during wall contact and maximize propulsion during push-off and underwater phases, thereby contributing substantially to overall race performance. The strong association observed between turn velocity and Aqua Points further reinforces the practical importance of turn-specific training in youth swimming.

The swimming efficiency index also differed significantly between groups, supporting the role of technical economy in swimming performance. Previous studies have shown that efficiency is closely related to improved coordination, reduced intracyclic velocity fluctuations, and optimized force application [1,21]. In youth swimmers, these improvements are typically associated with better body alignment and temporal synchronization of limb actions rather than increases in metabolic power [13,14].

Interestingly, significant differences were also observed in stroke count and stroke length between competitive levels. This finding partially contrasts with the initial hypothesis and suggests that, even during preadolescence, structural characteristics of the stroke cycle may contribute to competitive differentiation. Previous studies have shown that more skilled swimmers often achieve greater propulsion through more effective stroke organization and optimized distance per stroke [25,26]. Therefore, although coordination and efficiency remain central determinants, the present results indicate that stroke mechanics should not be disregarded when evaluating performance in 11–12-year-old swimmers.

Beyond the between-group comparisons, the regression analysis identified 25 m freestyle velocity and turn velocity as the strongest independent predictors of competitive performance. This finding aligns with previous research highlighting the importance of propulsion and hydrodynamic efficiency in determining swimming velocity [1,2,5]. While lower-limb propulsion showed significant differences between groups, its independent contribution was attenuated in the regression model and was ultimately excluded from the final model, suggesting that its effect may be mediated through overall swimming velocity and coordination patterns. This interpretation is consistent with previous findings indicating that the contribution of the lower limbs, although relevant, is secondary to whole-body coordination and propulsion efficiency during front crawl swimming [22,23].

From a developmental perspective, the age range studied represents a period characterized by rapid neuromuscular development and high responsiveness to motor learning [11,12]. The present findings reinforce the importance of prioritizing technical training during this stage, as performance gains appear to be strongly linked to improvements in coordination, movement efficiency, and technical execution.

From an applied standpoint, these results suggest that training programs for swimmers aged 11–12 years should focus on: (i) optimizing turn mechanics; (ii) enhancing sprint-specific propulsion; (iii) improving intersegmental coordination; and (iv) developing effective kicking technique within an integrated movement pattern. In addition, given the significant differences observed in stroke count and stroke length, coaches should also consider refining stroke organization and propulsion timing, ensuring that technical interventions promote both efficiency and effective stroke mechanics.

The absence of a significant Relative Age Effect indicates that, within this sample, competitive performance was not strongly influenced by birth quarter. However, the inclusion of this variable allowed for a more comprehensive analysis of potential developmental influences. Similarly, the inclusion of sex as a covariate highlights the importance of accounting for biological differences during this stage of development, where maturation rates may vary considerably [27].

A notable contribution of this study lies in linking an objective performance classification system (Aqua Points) with field-based biomechanical assessment. While scoring systems such as FINA Points have been widely used to compare performance levels [17], few studies have explored how these classifications relate to specific technical determinants in young swimmers. While Rudolph Points may be more sensitive to age-related performance differences, the use of Aqua Points in this study allowed us to focus on absolute competitive performance, which is strongly aligned with technical efficiency and race execution.

The present findings provide evidence that competitive level is closely associated with measurable biomechanical variables, supporting the use of field-based testing for performance monitoring and talent development.

Although the present study focused on biomechanical variables, swimming performance should be understood as multifactorial. Previous research has shown that psychosocial and environmental factors may also influence performance development [28,29]. For example, structured psychosocial interventions have been shown to enhance perceived support and training environment in youth swimmers [30,31]. These contextual factors may indirectly influence technical development and should be considered within a holistic athlete-development framework.

A key strength of this study is the relatively large sample of competitive swimmers assessed under standardized field conditions, enhancing both statistical robustness and ecological validity. However, several limitations should be acknowledged. First, the cross-sectional design does not allow causal inferences. Second, biological maturation was not directly assessed, which may influence performance variability at this age [11,15,16]. Third, the focus on short-distance performance limits the generalizability of the findings to longer events. Future research should incorporate longitudinal designs and direct measures of maturation to better understand developmental trajectories.

## 5. Conclusions

Competitive level in 11–12-year-old swimmers is strongly associated with short-distance swimming velocity, turn performance, swimming efficiency, and selected stroke mechanics. Among these factors, 25 m freestyle velocity and turn velocity emerged as the strongest predictors of competitive performance, highlighting their central role in sprint swimming success at this developmental stage.

These findings emphasize the importance of technical proficiency during preadolescence, where performance improvements appear to be driven by coordinated interactions between movement efficiency, propulsion, and stroke organization. From an applied perspective, training programs should prioritize the optimization of sprint velocity, turn execution, and technical efficiency while also refining stroke mechanics to enhance competitive performance in young swimmers.

## Figures and Tables

**Table 1:** Descriptive statistics (mean ± standard deviation) for performance and biomechanical variables across swimmers grouped according to Aqua Points classification.

Variable	Low (<224)	Medium (225–277)	High (>278)
Age (years)	11.5 ± 0.7	11.8 ± 0.6	12.0 ± 0.5
25 m Time (s)	17.5 ± 1.2	15.4 ± 0.8	14.7 ± 0.5
25 m Velocity (m·s^−1^)	1.44 ± 0.08	1.63 ± 0.07	1.72 ± 0.05
Kick Velocity (m·s^−1^)	0.95 ± 0.10	1.08 ± 0.09	1.18 ± 0.07
Turn Time (s)	7.35 ± 0.45	6.45 ± 0.40	5.75 ± 0.35
Turn Velocity (m·s^−1^)	1.36 ± 0.09	1.55 ± 0.08	1.74 ± 0.07

Note: Significant differences are reported in the text and visually indicated in Figure 2.

**Table 2:** Results of the stepwise multiple linear regression model predicting competitive performance (Aqua Points).

Variable	B	SE	t	*p*	Significance
(Constant)	−566.33	54.84	−10.33	<0.001	***
25 m Freestyle Velocity (m·s^−1^)	358.20	62.06	5.77	<0.001	***
Turn Velocity (m·s^−1^)	145.65	40.18	3.63	0.001	***
Efficiency Index (a.u.)	−25.04	11.11	−2.25	0.028	*
Sex (Female = 1)	86.67	9.56	9.06	<0.001	***

Note. B = unstandardized coefficient; SE = standard error; * *p* < 0.05; *** *p* < 0.001.

## Data Availability

The data presented in this study are available on request from the corresponding author.

## References

[1] Barbosa T.M., Bragada J.A., Reis V.M., Marinho D.A., Carvalho C., Silva A.J. (2010). Energetics and biomechanics as determining factors of swimming performance: Updating the state of the art. J. Sci. Med. Sport.

[2] Toussaint H.M., Beek P.J. (1992). Biomechanics of competitive front crawl swimming. Sports Med..

[3] Zamparo P., Pendergast D.R., Mollendorf J., Termin A., Minetti A.E. (2005). An energy balance of front crawl. Eur. J. Appl. Physiol..

[4] Kolmogorov S.V., Duplishcheva O.A. (1992). Active drag, useful mechanical power output, and hydrodynamic force coefficient in different swimming strokes at maximal velocity. J. Biomech..

[5] Sorgente V., Agudo-Ortega A., Lopez-Hernandez A., Santos del Cerro J., Minciacchi D., González Ravé J.M. (2023). Relationship between Maximum Force–Velocity Exertion and Swimming Performances among Four Strokes over Medium and Short Distances: The Stronger on Dry Land, the Faster in Water?. J. Funct. Morphol. Kinesiol..

[6] Arellano R., Brown P., Cappaert J., Nelson R.C. (1994). Analysis of 50-, 100-, and 200-m freestyle swimmers at the 1992 Olympic Games. J. Appl. Biomech..

[7] Blanksby B.A., Nicholson L.L., Elliott B. (2002). Swimming: Biomechanical analysis of the grab, track and handle swimming starts: An intervention study. Sport Biomech..

[8] Veiga S., Roig A. (2016). Underwater and surface strategies of 200 m world-level swimmers. J. Sports Sci..

[9] Lyttle A.D., Mason B.R. (1997). A kinematic and kinetic analysis of the freestyle and butterfly turns. J. Sci. Med. Sport.

[10] Veiga S., Roig A. (2017). Effect of the starting and turning performances on the subsequent swimming parameters of elite swimmers. Sports Biomech..

[11] Malina R.M., Bouchard C., Bar-Or O. (2004). Growth, Maturation, and Physical Activity.

[12] Lloyd R.S., Oliver J.L. (2012). The youth physical development model: A new approach to long-term athletic development. Strength. Cond. J..

[13] Morais J.E., Silva A.J., Marinho D.A., Lopes V.P., Barbosa T.M. (2017). Determinant factors of long-term performance development in young swimmers. Int. J. Sports Physiol. Perform..

[14] Figueiredo P., Zamparo P., Sousa A., Vilas-Boas J.P., Fernandes R.J. (2011). An energy balance of the 200 m front crawl race. Eur. J. Appl. Physiol..

[15] McNarry M.A., Lester L., Brown J., Mackintosh K.A. (2020). Investigating the Modulatory Role of Chronological and Biological Age on Performance Predictors in Youth Swimmers. J. Sci. Sport Exerc..

[16] Almeida-Neto P., de Assis G.G., Silva B.D.C., Medeiros R., Bulhões-Correia A., Oliveira V., de Queiros V.S., De Paiva P., Dantas P.M.S., Cabral B. (2022). Contribution of biological maturation and power of upper and lower limbs to crawl swimming performance in young swimmers. Hum. Mov..

[17] World Aquatics. Swimming Points. https://www.worldaquatics.com/swimming/points.

[18] Santos C.C., Garrido N.D., Cuenca-Fernández F., Marinho D.A., Costa M.J. (2023). Performance Tiers within a Competitive Age Group of Young Swimmers Are Characterized by Different Kinetic and Kinematic Behaviors. Sensors.

[19] Neiva H.P., Marques M.C., Barbosa T.M., Izquierdo M., Marinho D.A. (2014). Warm-up and performance in competitive swimming. Sports Med..

[20] Cuenca-Fernández F., Boullosa D., López-Belmonte Ó., Gay A., Ruiz-Navarro J.J., Arellano R. (2022). Swimming Warm-Up and Beyond: Dryland Protocols and Their Related Mechanisms-A Scoping Review. Sports Med. Open..

[21] Pyne D.B., Sharp R.L. (2014). Physical and energy requirements of competitive swimming events. Int. J. Sport Nutr. Exerc. Metab..

[22] Seifert L., Toussaint H.M., Alberty M., Schnitzler C., Chollet D. (2010). Arm coordination, power, and swim efficiency in national and regional front crawl swimmers. Hum. Mov. Sci..

[23] Gatta G., Cortesi M., Di Michele R. (2012). Power production of the lower limbs in flutter-kick swimming. Sports Biomech..

[24] Morouço P.G., Marinho D.A., Izquierdo M., Neiva H., Marques M.C. (2015). Relative Contribution of Arms and Legs in 30 s Fully Tethered Front Crawl Swimming. Biomed. Res. Int..

[25] Morais J.E., Marinho D.A., Arellano R., Barbosa T.M. (2019). Start and turn performances of elite sprinters at the 2016 European Championships in swimming. Sports Biomech..

[26] Craig A.B., Pendergast D.R. (1979). Relationships of stroke rate, distance per stroke, and velocity in competitive swimming. Med. Sci. Sports..

[27] Seifert L., Chollet D., Chatard J.C. (2007). Kinematic changes during a 100-m front crawl: Effects of performance level and gender. Med. Sci. Sports Exerc..

[28] Matjiur R., Chainok P., Lauer J., Limroongreungrat W., de Jesus K., Zacca R., Fernandes R.J., Vilas-Boas J.P. (2025). Sex-based differences in front crawl and butterfly sprint performance in age-group swimmers. PLoS ONE.

[29] Araújo D., Davids K., Hristovski R. (2006). The ecological dynamics of decision making in sport. Psychol. Sport Exerc..

[30] Davids K., Glazier P., Araújo D., Bartlett R. (2003). Movement systems as dynamical systems: The functional role of variability and its implications for sports medicine. Sports Med..

[31] López-Hernández A., Simón-Piqueras J.Á., Zamorano-García D., Pyne D.B., Ravé J.M.G. (2025). Impact of psychosocial intervention on performance determinants in competitive swimmers: Roles of coach, family, environment, and athlete characteristics. Sports.

